# An air insufflation device for reduction of intussusception in children

**DOI:** 10.4103/0971-9261.43794

**Published:** 2008

**Authors:** Reju J. Thomas, Syam Rakhesh

**Affiliations:** Department of Paediatric Surgery, Kerala Institute of Medical Sciences, Trivandrum, Kerala, India; 1 Department of Biomedical Engineering, Kerala Institute of Medical Sciences, Trivandrum, Kerala, India

**Keywords:** Air enema, child, intussusception, pneumatic reduction

## Abstract

The authors have developed a portable device for insufflation of air reliably at pressures accepted as safe for effective reduction of intussusception in children under fluoroscopic guidance. The results of reduction with the device were equal to those by saline enema reduction under ultrasound guidance.

## INTRODUCTION

**Figure 1:**
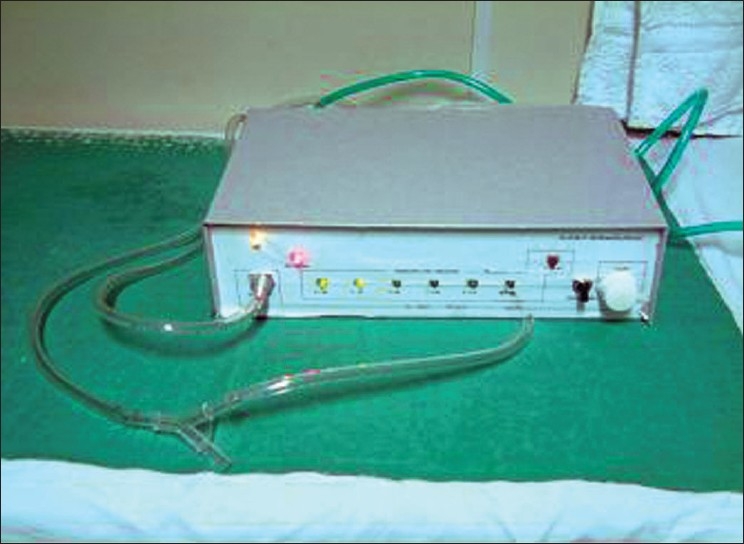
Air insufflation device for reduction of intussusception

Most intussusceptions in children are idiopathic and majority can be reduced safely without recourse to surgery. All the three methods i.e. hydrostatic reduction with a barium saline enema under fluoroscopic guidance, hydrostatic saline reduction under ultrasound guidance and air insufflation under fluoroscopic guidance – have equal success rates. The concerns of missing bowel pathology, reduction of gangrenous bowel with consequent shock, perforation of bowel and causing peritonitis have been adequately addressed by adopting criteria for case selection and delineation of pressures of 80–130 mmHg as effective and safe for reduction of intussusception.[1,2] Of the several elements in this technique, we were particularly troubled by the soiling and spillage around the catheter in hydrostatic enemas and leakage of air with air enema that made it difficult for us to maintain the desired pressure. Soiling of the radiology suite was no longer a problem once we adopted air enema. Several simple devices have been described for easy performance of air enema.[3,4] With an oxygen cylinder pressure reducer, flow meter and a manual spring-loaded pressure release valve, leakage continued to be a problem, which was usually managed by increasing the rate of insufflation. It remained difficult to control the pressure and the child, and to monitor the progress of reduction at the same time. With these problems in mind we proceeded to make a portable insufflation device that would deliver air at rates sufficient to maintain pressure without the danger of pressure overshoot [Figure 1].

## MATERIALS AND METHODS

A literature search using Medline search in English from 1996 for air enema reduction of intussusception and its complications did not yield any reports of air embolism when atmospheric air was used. Reports of perforation identified those predisposed to perforation as having one of the following: shock, peritonism, gross abdominal distension and air-fluid levels on abdominal X-ray and attempt at air enema after operative reduction. Pressures considered safe for use ranged from 60 to 130 mm Hg. If and when perforation did occur, the pneumoperitoneum could be easily recognised[5]. Experimentally and clinically, fecal contamination was less with air enema perforation when compared with perforation during a hydrostatic enema.[5,6]

In the author’s practice of pneumatic reduction using oxygen through a flowmeter from an oxygen cylinder and a handheld anaeroid manometer, the flow rate required for reduction when using a standard technique of an 18F Foley’s catheter and perianal strapping was in the range of 2–6 l/min. The device specifications were laid down accordingly.

An electrically operated pump was combined with a solenoid valve to vent air when the pressure exceeded the preset value. A rotary knob to dial up preset pressures in the range of 80–130 mm Hg, a battery backup and a reservoir to act as a condenser to smooth out air delivery and pressure fluctuations were added. An additional inlet was subsequently added to the device in case there was a need to augment the air supply in special instances. Calibration of the device was performed to meet specifications on air flow, to ensure that the pressures dialled up were within 2mm Hg of intended values and that the solenoid blow-off valve could vent up to 12 l/min of air flow to prevent pressure overshoot. The device was then put into regular clinical practice.

## RESULTS

In our department, indications for laparotomy in childhood intussusceptions are toxemia, abdominal distension, an age range outside of 3 months to 3 years and intussusception on its second recurrence. In the others, nonoperative reduction was attempted with air enema and saline enema based on consultant preference. In an audit for the Department of Paediatric Surgery for the year 2007, the success rate for ultrasound-guided saline reduction was 85.3% (29 out of 34 instances), with no early recurrences. Within the same period, the success rate for air enema reduction with the new device was 88.2% (15 out of 17 instances). One of this was in a 2-year-old who had recurrence of intussusceptions 8 months after a successful saline reduction in the previous calendar year. There was one early recurrence in 24 h that was reduced successfully by a repeat air enema. In addition, air enema was used to achieve partial reduction in one child with established intestinal obstruction to help in making the incision in the right iliac fossa and reduced manipulation required at laparotomy.

## DISCUSSION

The air insufflations device was developed to meet the needs of an air enema. Its unique features are: It is portable, with the electrical pump working on a rechargeable battery that can be charged in between procedures. The pressures delivered are accurate within a narrow range. A reservoir blunts pressure fluctuations and a pressure release valve blows off excess pressure that develops due to bowel contractions or straining. The flow rates required were determined in a pilot study and are adequate to achieve reduction even in the face of moderate air leak. An audible pressure drop alarm alerts the operator to a significant air leak in the system. LED lights provide a visual confirmation of the pressure used in the low-lighting environment of the fluoroscopy room.

Oxygen is not required and the device does not represent an explosion hazard. The device does not contain mercury or glass that can spill or shatter to cause injury to personnel.

With this portable device, a Foley’s catheter, a 20 ml syringe and adhesive tape are the only materials that are necessary to initiate an air enema. This reduces the set-up time. Air enema typically takes 1–3 min of the fluoroscopy time.[6] This reduces the procedure time during which the child is distressed. These, along with the absence of spillage, provide for a rapid turnaround time for the radiology suite.
